# One Hundred Years of Hypertension Research: Topic Modeling Study

**DOI:** 10.2196/31292

**Published:** 2022-05-18

**Authors:** Mustapha Abba, Chidozie Nduka, Seun Anjorin, Shukri Mohamed, Emmanuel Agogo, Olalekan Uthman

**Affiliations:** 1 Warwick Centre for Global Health, Division of Health Sciences University of Warwick Medical School University of Warwick Coventry United Kingdom; 2 Country Office Nigeria Resolve to Save Lives Abuja Nigeria

**Keywords:** hypertension, high blood pressure, machine learning, topic modeling, latent Dirichlet allocation, LDA, cardiovascular, research trends

## Abstract

**Background:**

Due to scientific and technical advancements in the field, published hypertension research has developed substantially during the last decade. Given the amount of scientific material published in this field, identifying the relevant information is difficult. We used topic modeling, which is a strong approach for extracting useful information from enormous amounts of unstructured text.

**Objective:**

This study aims to use a machine learning algorithm to uncover hidden topics and subtopics from 100 years of peer-reviewed hypertension publications and identify temporal trends.

**Methods:**

The titles and abstracts of hypertension papers indexed in PubMed were examined. We used the latent Dirichlet allocation model to select 20 primary subjects and then ran a trend analysis to see how popular they were over time.

**Results:**

We gathered 581,750 hypertension-related research articles from 1900 to 2018 and divided them into 20 topics. These topics were broadly categorized as preclinical, epidemiology, complications, and therapy studies. Topic 2 (*evidence review*) and topic 19 (*major cardiovascular events*) are the key (*hot topics*). Most of the cardiopulmonary disease subtopics show little variation over time, and only make a small contribution in terms of proportions. The majority of the articles (414,206/581,750; 71.2%) had a negative valency, followed by positive (119, 841/581,750; 20.6%) and neutral valency (47,704/581,750; 8.2%). Between 1980 and 2000, negative sentiment articles fell somewhat, while positive and neutral sentiment articles climbed substantially.

**Conclusions:**

The number of publications has been increasing exponentially over the period. Most of the uncovered topics can be grouped into four categories (ie, preclinical, epidemiology, complications, and treatment-related studies).

## Introduction

Hypertension, together with its repercussions, has been identified as a substantial public health concern. In recent years, there has been major growth in global hypertension research activities [1,2]. Growing worries about the global epidemic of hypertension as well as the need to offer information to policy makers and decision makers on better prevention, treatment, and control have contributed to this growth [3]. Hypertension is a global health problem, according to the World Health Organization, with an estimated one billion people having hypertension [4]. Hypertension-related diseases such as stroke and ischemic heart disease are among the leading causes of death worldwide. As a result, putting in place evidence-based prevention, early detection, and long-term control methods that reduce mortality and improve quality of life is unquestionably necessary.

There are a lot of studies on hypertension that have been published [1]. Reading and interpreting all of these publications using typical information retrieval and review methods would be time-consuming and labor-intensive, if not impossible. Finding more delicate themes and comprehending the complicated links between such publications is considerably more difficult, but it is necessary to determine whether or not studies are consistent. Machine learning–based literature mining enables the analysis of large collections of documents in a highly automated manner [5,6]. Model topic algorithms can uncover hidden or latent subjects from large document collections automatically [7]. Documents may speak for themselves by the unattended nature of latent Dirichlet allocation, and subjects emerge without human intervention [8]. The study aims to generate evidence on the evolution of hypertension research that could ultimately inform prioritization of research, financial investments, and health policy. The aim of the study was to use natural language processing techniques to provide overview of hypertension research topics over 100 years.

## Methods

### Methods Overview

We searched PubMed to obtain the records of all hypertension articles published in the last 100 years using the following search strategy: “(“hypertension”[MeSH Terms] OR “hypertension”[All Fields] OR (“high”[All Fields] AND “blood”[All Fields] AND “pressure”[All Fields]) OR “high blood pressure”[All Fields]). The title and abstract of each article were extracted and combined into a single string.

### Phase 1: Preprocessing

To create a document-term matrix, each article was tokenized (divided) into a list of terms (words). Text was filtered to exclude common keywords with no analytical significance (prepositions, articles, pronouns), punctuations, and digits. Following that, stemming was done, which is the process of deleting frequent word ends (eg, “compression,” “compressed,” and “compressing” are converted to “compress”). The frequency of each word was normalized using the frequency of the most common word used in all articles that year, and a scale of 1 to 100 was produced (1 being most frequently used, 100 being least frequently used). The goal of normalization techniques like stemming and lemmatization is to reduce inflectional forms and sometimes derivationally related forms of a word to a common base form.

### Phase 2: Topic Extraction

The preprocessed document-term matrix was then subjected to latent Dirichlet allocation. Latent Dirichlet allocation is a hierarchical Bayesian algorithm and one of the most common topic modeling approaches. Latent Dirichlet allocation identifies theme subjects by looking for keywords that frequently appear together in a document. The model then uses the associations between terms to define two things: (1) themes that are each characterized by a distribution of words and (2) documents as a distribution of topics. As a result, latent Dirichlet allocation is well suited to assessing articles that cover a wide range of topics. Using a collapsed Gibbs sampler set to run for 5000 iterations, model parameters were provided to uncover 50 themes with high interpretability (Dirichlet hyperparameters: α=.02, η=0.02). After the model fitting was complete, topic numbers were allocated.

The probability distribution of words in each topic was then visualized and used to create word clouds. The top 15 most likely terms in each topic were then put into a word cloud, with greater font size and darker color indicating higher probability.

Topic popularity and dynamics were determined by dividing the total number of abstracts in each year by the cumulative sum of articles belonging to each topic, yielding a percentage of subjects in each year. The most popular themes in each article were also determined by assessing subject popularity by article. Using simple linear regression and Cochran-Armitage trend testing, a trend analysis was performed to identify themes with growing (“hot”) or decreasing (“cold”) popularity over time.

### Phase 3: Topic-Based Sentiment Analysis

The first step in the sentiment analysis was to align the preprocessed text with the valence classification (eg, positive, or negative). The Emotion Lexicon [9] is a database that indexes the valence and emotion of over 4000 regularly used English lemmas. Most words in the vocabulary are classified as positive or negative. By summing the counts of positive and negative terms, the overall positive and negative valence for each item was computed. The ratios were calculated by dividing the number of positive words in each article by the number of nonstop words, and vice versa for negative words (scores ranging from 0 neutral to 1 highest). The final score was expressed as a percentage of positive or negative words compared to other important words in the article.

### Ethics approval and consent to participate

This study was based on an analysis of existing data collected from PubMed.

## Results

### Trends in Hypertension Research

**Figure 1:**
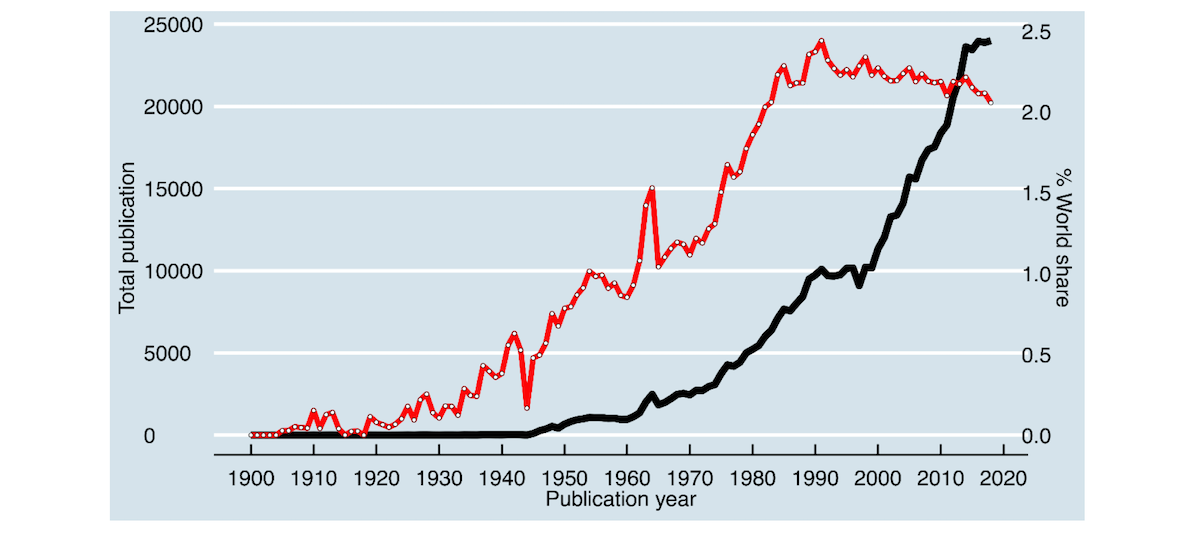
Trends in the number of hypertension research indexed in PubMed, 1900 to 2018.

A total of 581,750 articles on hypertension research were indexed between 1900 and 2018 in PubMed. As shown in Figure 1*,* the number of publications has been increasing exponentially over the period. The studied period was divided into the following three stages: the first stage ran from 1900 to 1940 (average publication: 7 per year), the second stage ran from 1941 to 1990 (3000 per year), and the third stage ran from 1991 to 2018 (15,000 per year). The period from 1991 to 2018 was a rapid development period, accounting for almost 75% of all hypertension research (434,487/581,750, 74.7%). Table 1 provides an overview of the 20 topics, the top 15 words of the topics with their probabilities, and the manually attached label that best captures the semantics of the words.

Most of the uncovered topics can be grouped into four categories (ie, preclinical, epidemiology, complications, and treatment-related studies). Topic 12, *animal model*, is most prevalent in the *preclinical studies* category. It contains salient words like *rat*, *inhibit*, *mice*, and *cell*. Topic 2, *evidence review* is most prevalent in the *risk factors studies* category. It contains salient words like *disease*, *review*, and *prevent*. Topic 19, *major cardiovascular events* is most prevalent in the *complications studies* category. It contains salient words like *stroke*, *coronary*, and *mortal*. Topic 10, *antihypertensive*, and topic 18, *heart surgery* are both about pharmacotherapy and interventional cardiology.

**Figure 2:**
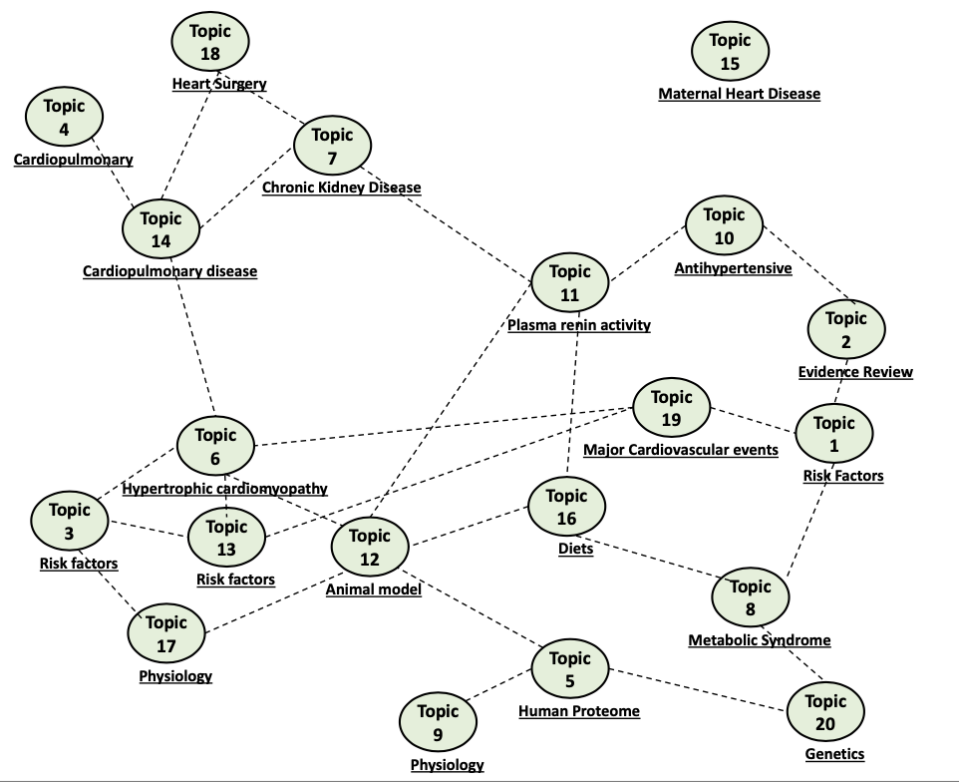
The clustering topics.

The clustering topics are shown in Figure 2, connected topics based on the similarity of topic probability distributions over the documents. The topics that were more likely included in the same articles had a high level of similarity in distribution of topics over the documents and thus were paired or clustered together. Several interesting clusters can be seen. Topics 5 (*human proteome*), topic 9 (*physiology*), and topic 20 *genetic* are paired, which means these articles are focusing on the pathophysiology of hypertension. Another example is topic 11 (*plasma renin activity*), topic 7 *chronic kidney disease*), and topic 18 (*heart surgery*) was often discussed in the same articles. It is important to note that topic 15 *maternal heart disease* is the only topic not connected to other topics (ie, not usually discussed in the same article with other topics).

**Figure 3:**
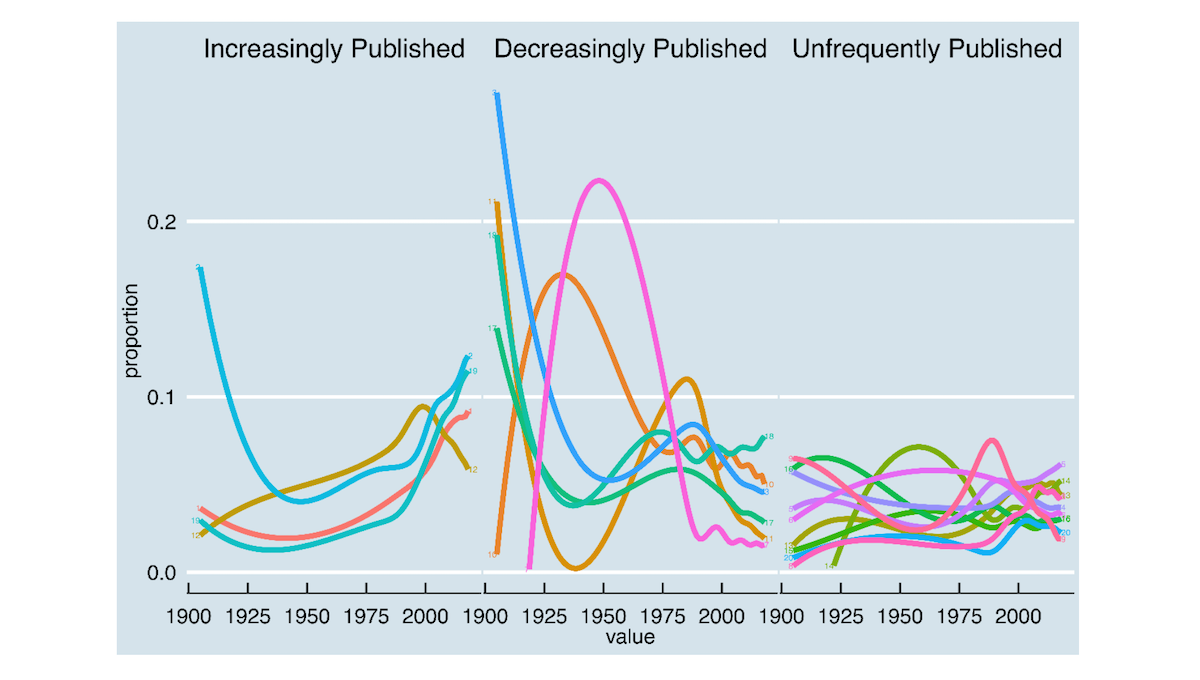
Dynamics and trends of the topics.

Figure 3 shows the temporal dynamics of the distributions of all topics. It demonstrates how the popularity of each topic has changed relative to other topics over time. The interpretation of these trends is speculative, but three categories of interest were identified: increasingly *hot* (topics 1, 2, 12, and 19), decreasingly *cold* (topics 3, 7, 10, 11, 17, and 18), and infrequently published topics (topics 4, 5, 6, 8, 9, 13, 14, 15, 16, and 20). Topic 2, *evidence review* and topic 19 *major cardiovascular events* are the key *hot topics*. Most of the cardiopulmonary disease subtopics show little variation over time, and only make a small contribution in terms of proportions.

**Table 1:** Topic classification and keywords on hypertension.

Classification and topic name		Keywords	Articles, n (%)
**Preclinical studies**			
	Topic 5: human proteome	cell, protein, human, activ, tissu, express, use, studi, factor, show, membran, model, peptid, antibodi, tumor	23,270 (4.0)
	Topic 9: biochemical	plasma, method, use, concentr, sampl, determin, liquid, detect, extract, chromatographi, acid, human, metabolit, pharmacokinet, serum	32,578 (5.6)
	Topic 11: plasma renin activity	plasma, hypertens, effect, level, increas, signific, activ, renin, concentr, blood, urinari, calcium, excret, serum, decreas	27,342 (4.7)
	Topic 12: animal model	rat, hypertens, increas, activ, receptor, vascular, effect, express, oxid, shr, inhibit, respons, mice, role, cell	37,814 (6.5)
	Topic 17: physiology	brain, respons, sympathet, rate, increas, activ, heart, effect, pressur, group, control, nerv, hypotens, system, signific	19,780 (3.4)
	Topic 20: genetics	gene, hypertens, associ, genet, polymorph, studi, genotyp, famili, mutat, allel, signific, variant, control, analysi, phenotyp	11,635 (2.0)
**Epidemiology**			
	Topic 1: risk factors	risk, age, studi, associ, factor, hypertens, preval, women, blood, year, men, among, level, cholesterol, high	37,814 (6.5)
	Topic 2: evidence review	diseas, hypertens, use, clinic, care, review, health, medic, manag, cardiovascular, patient, prevent, includ, studi, treatment	58,175 (10.0)
	Topic 3: blood pressure measurement	pressur, blood, measur, flow, arteri, exercis, mmhg, hypertens, increas, mean, chang, use, differ, signific, studi	33,742 (5.8)
	Topic 13: correlation studies	hypertens, patient, group, arteri, signific, systol, pressur, function, diastol, age, subject, correl, index, left, studi	22,107 (3.8)
	Topic 16: diets	diet, intak, blood, sodium, dietari, salt, group, pressur, effect, vitamin, hypertens, weight, increas, high, acid	14,544 (2.5)
**Complications**			
	Topic 4: cardiopulmonary	group, transplant, oxygen, patient, lung, acut, increas, dialysi, injuri, level, ventil, signific, high, pulmonari, respiratori	17,453 (3.0)
	Topic 6: hypertrophic cardiomyopathy	cardiac, heart, ventricular, left, myocardi, coronari, failur, hypertrophi, right, arteri, increas, atrial, group, infarct, function	18,616 (3.2)
	Topic 7: chronic kidney disease	renal, kidney, arteri, diseas, patient, function, portal, chronic, caus, children, stenosi, adren, treatment, case, progress	30,833 (5.3)
	Topic 8: metabolic syndrome	metabol, diabet, syndrom, insulin, obes, glucos, level, type, associ, resist, sleep, patient, increas, met, serum	19,780 (3.4)
	Topic 14: cardiopulmonary disease	pulmonari, patient, hypertens, arteri, diseas, pah, lung, sever, heart, clinic, right, valv, transplant, associ, vascular	25,597 (4.4)
	Topic 15: maternal heart disease	women, pregnanc, hypertens, matern, gestat, infant, birth, fetal, preeclampsia, deliveri, neonat, studi, pregnant, outcom, group	14,544 (2.5)
	Topic 19: major cardiovascular events	patient, risk, diseas, factor, associ, diabet, studi, stroke, age, year, mortal, coronari, cardiovascular, incid, use	40,723 (7.0)
**Treatment**			
	Topic 10: antihypertensive	patient, treatment, hypertens, effect, therapi, drug, group, blood, pressur, studi, antihypertens, trial, control, combin, signific	45,958 (7.9)
	Topic 18: heart surgery	patient, case, hypertens, surgeri, present, complic, report, surgic, portal, clinic, year, postop, vein, one, intracrani	49,449 (8.5)

### Article Sentiment Analysis

**Figure 4:**
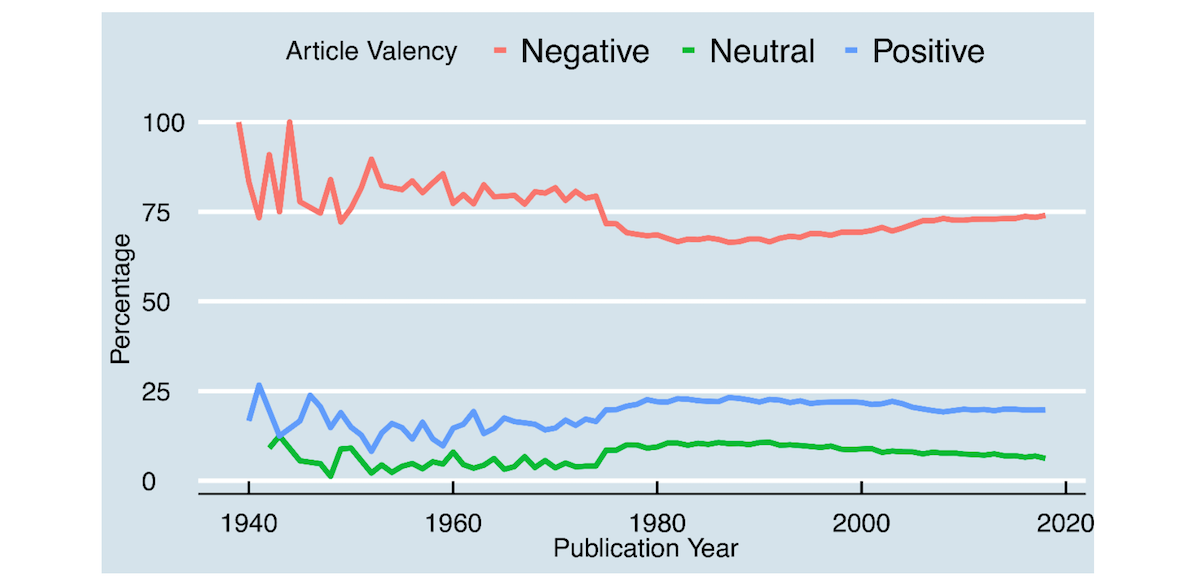
Dynamics and trends of the article valency.

Most of the articles were framed with a negative valency (414,206/581,750; 71.2%), followed by positive (119, 841/581,750; 20.6%) and neutral valences (47,704/581,750; 8.2%). Yearly sentiment trends with hypertension articles are shown in Figure 4.

Negative sentiments articles decreased slightly between 1980 and 2000. While both positive and neutral sentiments articles increased slightly over the same period. The top 20 frequently used positive and negative words are shown in Multimedia Appendix 1.

*Risk*, *chronic*, *syndrome*, *failure*, and *severe* were the more common negative sentiments words, whereas *healthy*, *effective*, *positive*, *survive*, and *improved* were the more common positive sentiments words.

## Discussion

### Principal Findings

In this study found that more than half a million articles have been published on hypertension worldwide between 1900 and 2018. We identified 20 distinct topics, which can be categorized broadly into preclinical, epidemiology, complications, and therapy studies. We used an unsupervised text mining methodology to find hypertension research topics and their dynamics in this study, which looked at publications indexed in PubMed during the last century. This study adds to our understanding of hypertension research focuses and their evolutionary patterns, and it may aid researchers, journal editors, and funders in identifying new or ignored trends from established themes, as well as freshly developing trends that can be evaluated in a structured way.

Our findings revealed that the majority of the topics discovered may be divided into four categories (ie, preclinical, risk factors, complications, and treatment-related studies). The important *hot* topics include topic 2 *evidence review* and topic 19 *major cardiovascular events*. The majority of the cardiopulmonary disease subtopics show little fluctuation over time and contribute a modest share of the total. In the eyes of the researcher, commonly published topics may represent a large body of knowledge, a common disorder, or a low-cost easy-to-study subject. On the other hand, less often published topics may represent the study of less common hypertension-related disorders and subject matter that is difficult or expensive to research. Some pathophysiological research, for example, necessitates a substantial investment of time and resources as well as collaboration among physicians, biochemists, and physiologists. Similarly, some genetic disorders may be highly rare and have a high unmet demand, posing substantial therapy and research obstacles.

In the themes, we saw some intriguing clumping. Topics 5 (*human proteome*), 9 (*physiology*), and 20 (*genetic*) were paired, indicating that these papers were all on hypertension pathophysiology. Topics 11 (*plasma renin activity*), 7 (*chronic kidney illness*), and 18 (*heart surgery*), for example, were frequently mentioned in the same articles. It is worth noting that topic 15, *maternal heart disease*, is the only one that is not linked to the others because it is not mentioned in the same article.

### Comparison to Prior Work

Although more than half a million hypertension research articles have been published in the past 100 years, only a few bibliometric articles have summarized the development and application of hypertension [10-12]. To better understand the development status, research hot spots and future development trends of hypertension, it is necessary to conduct a comprehensive retrospective analysis. In recent years, hypertension research has developed rapidly, and the amount of publications has increased exponentially. This may be due to the attention paid to the burgeoning burden of high blood pressure in various countries to promote the widespread use of prevention and prompt treatment. Bibliometric analysis is an extremely useful tool despite its focus on international peer-reviewed journals; therefore, together with our theoretical and methodological approaches taken to identify relevant global publications and the data analysis used, we have a strong base on which to state that our study presents a comprehensive systematization of global hypertension research.

### Implications for Practice and Further Research

This bibliometric analysis provides insight into the historical development of hypertension research. Scientific publications play an important role in the scientific process providing a key linkage between knowledge production and use. These data reveal a solid mass of research activities on hypertension. This study provides useful information to researchers and funding societies concerned in the implementation of research strategies to improve hypertension research. Additionally, the results of this study delineate a framework for better understanding the situations of current hypertension research and prospective directions of the research in this field that could be applied for managing and prioritizing future research efforts in hypertension research. Our study provides some novel insights useful for policy makers, researchers, and funders interested in advancing hypertension research agenda. International research collaborations and research networks should be encouraged to help prioritize hypertension research particularly in women. Our findings provide baseline data for scholars and policy makers to recognize the bibliometric indicators in this study as measures of research performance in hypertension for future policies and funding decisions. Finally, our study showed that bibliometric analysis is a good methodological tool to map published literature in a particular subject and to pinpoint research gaps in that subject.

Although topic modeling has previously been used to analyze medication safety research trends [13], we believe this is the first study to apply unsupervised machine learning to assess hypertension research subjects and patterns over the past century. The procedure of data analysis was rather objective. However, the majority of the papers included in the database were written in English, which means that relevant studies published in other languages may have been overlooked.

### Strengths and Limitations

Our study had various advantages, including a thorough examination of hypertension research from a variety of medical specialties. Our study has a lot of limitations. For starters, we only looked at articles, reviews, and editorials published in academic journals indexed in PubMed as part of the study design. As a result, this research does not claim to represent all of the work done on this issue, some of which may have been published in other formats (eg, books, reports, and national journals). We also do not pretend to give exact numbers in terms of country contributions to global scientific production because we did not hand-search all of the retrieved papers to ensure their relevance, though we believe our findings reflect broad trends in the hypertension research environment.

Furthermore, the primary source of this bibliometric analysis was international academic journals indexed in PubMed, and international journals are known to contain an English language bias, which may skew our results in favor of Anglo-Saxon countries or countries where the national research system encourages publishing primarily in these types of journals [14,15]; some non–Anglo-Saxon countries have national research systems that encourage and prioritize national publications. Despite bibliometric databases expanding their journal coverage, this may diminish the international awareness of the study and obscure the true volume of research undertaken in these countries. Scholars have speculated whether *editorial racism* exists in the evaluation and selection of manuscripts for publication in international journals, with prejudice against authors from the Global South, and Harris et al [16] showed (and measured) bias by health professionals and researchers against low-income countries’ research compared to high-income countries’ research [17,18]. Nonetheless, rising investment in research in the Global South that includes a greater emphasis on good methodology, research infrastructure, and high-quality presentation in terms of both writing and (English) language skills could potentially offset such peer prejudice [17].

While our findings are based solely on publications in international academic journals, they are important to consider because of the importance placed on publishing in international academic journals in academia, and how it is frequently used to inform decisions about international development, policy, and research agendas. Furthermore, our findings are likely to allude to the global dynamic within this field of study. Quantitative bibliometric results say nothing about the quality of research conducted in countries worldwide; more research is needed to contextualize our findings and provide in-depth insights into the types of theoretical and methodological approaches being used and where, as well as national research priorities, and to enrich the current understanding of the historical and structural determinants of global bibliometric trends and inequity. Finally, it is important to note that common lexicons for sentiment analysis have many limitations when applied to health literature. For example, “negative” terms in the used lexicon likely are not negative in scientific literature (eg, symptoms or inhibition), and some “positive” labels (eg, survival, advanced, and progressive) are more likely to have negative sentiment in hypertension-based literature.

In this publication, we report an empirical analysis that used latent Dirichlet allocation modeling to identify key research themes based on research published in hypertension publications. We also looked at the themes’ dynamics and intellectual structure. The findings gave a complete overview of hypertension-related research subjects and highlighted how these issues have evolved over time. The findings of this study could help us better understand hypertension research trends and propose areas for further study.

## Appendix

Multimedia Appendix 1 Top 20 negative and positive words.

## Abbreviations

PTDF: Petroleum Technology Development Fund

## Data Availability

The data are publicly available. All data generated or analyzed during this study are included in this paper. The analysis was based on data sets collected from PubMed. Information on the data and content can be accessed in PubMed.
