# Supplementary figures and images for: One Hundred Years of Hypertension Research: Topic Modeling Study
Source: JMIR Form Res. 2022 May 18;6(5):e31292. doi: 10.2196/31292 (PMC9161044; doi:10.2196/31292)

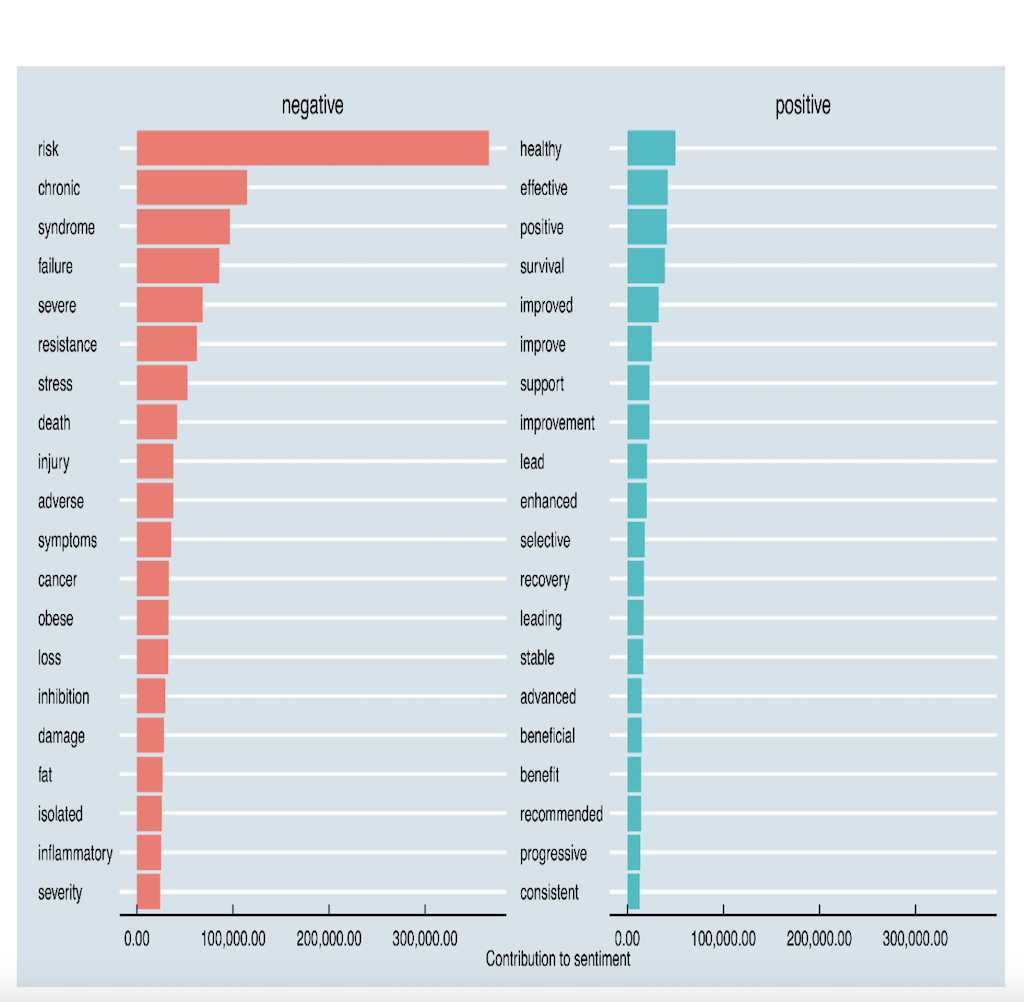

Supplement: Multimedia Appendix 1 [file formative_v6i5e31292_app1.png]
